# The German S3-level guideline for the diagnosis and management of rare dental disorders

**DOI:** 10.1007/s00784-026-07142-w

**Published:** 2026-09-22

**Authors:** Helena Dujic, Katharina Bücher, Ina Manuela Schüler, Peter Schmidt, Susann Hertel, Julia Timpel, Anahita Jablonski-Momeni, Reinhard Schilke, Ines Kapferer-Seebacher, Johannes Zschocke, Anja Liebermann, Jan Frederik Güth, Daniel Edelhoff, Roswitha Heinrich-Weltzien, Jan Kühnisch

**Affiliations:** 1 Conservative Dentistry, Periodontology and Digital Dentistry, University Hospital, LMU Munich, Munich, Germany; 2 Department of Orthodontics, Section of Preventive and Pediatric Dentistry, Jena University Hospital, Friedrich-Schiller-University of Jena, Jena, Germany; 3 Department of Conservative Dentistry and Periodontology, Centre of Dentistry, Oral and Maxillofacial Medicine, Ulm University Hospital, University of Ulm, Ulm, Germany; 4 Clinic of Operative and Pediatric Dentistry, Medical Faculty Carl Gustav Carus, Dresden University of Technology, Dresden, Germany; 5 Department of Orthodontics, Dental School, Philipps-University Marburg, Marburg, Germany; 6 Department of Conservative Dentistry, Periodontology and Preventive Dentistry, Hannover Medical School, Hannover, Germany; 7 University Hospital for Conservative Dentistry and Periodontology, Medical University of Innsbruck, Innsbruck, Austria; 8 Division of Human Genetics, Medical University of Innsbruck, Innsbruck, Austria; 9 Department of Prosthetic Dentistry, University Hospital of Cologne, Cologne, Germany; 10 Department of Prosthetic Dentistry, University Hospital, LMU Munich, Munich, Germany; 11 Ludwig-Maximilians-Universität München Poliklinik für Zahnerhaltung, Parodontologie und digitale Zahnmedizin Goethestraße 70, 80336 München, Germany

**Keywords:** Amelogenesis imperfecta, Dentinogenesis imperfecta, Ectodermal dysplasia, X-linked hypophosphatemia, Hypophosphatasia, Periodontitis, Rare systemic disease

## Abstract

**Objectives:**

The aim of this German S3-level guideline project was to develop evidence-based recommendations to support professionals in the diagnosis and management of individuals with rare dental disorders. The research question was which form of dental care should be part of an age-related, long-term treatment for individuals affected by amelogenesis imperfecta, dentinogenesis imperfecta, ectodermal dysplasia, X-linked hypophosphatemia, hypophosphatasia or rare periodontal disorders.

**Materials and methods:**

The guideline development combines a systematic search of the literature, expert input from multiple disciplines and a structured consensus process. In addition to phrasing recommendations on clinical, radiological and genetic diagnostics, much emphasis has been placed on the development of disease-specific treatment recommendations. This article presents a summary of the German guideline.

**Results:**

A total of 41 evidence- and consensus-based recommendations covering interdisciplinary collaboration, diagnostics, preventive care, restorative and prosthetic treatment, and orthodontic and surgical interventions were developed. The recommendations emphasise the importance of tailoring dental care to the individual patient’s needs.

**Conclusions:**

For the first time, an evidence-based guideline is available for the dental management of individuals affected by rare dental disorders. The guideline provides practical guidance and highlights the need for interdisciplinary management approaches. Furthermore, the guideline establishes a foundation for standardised, evidence-based dental care.

**Clinical relevance:**

This guideline is expected to improve oral health outcomes and quality of life in this vulnerable patient group. Although developed in Germany, it may inform adoption or adaptation internationally, thereby fostering harmonised standards of care in different healthcare systems.

## Introduction

**Fig. 1:**
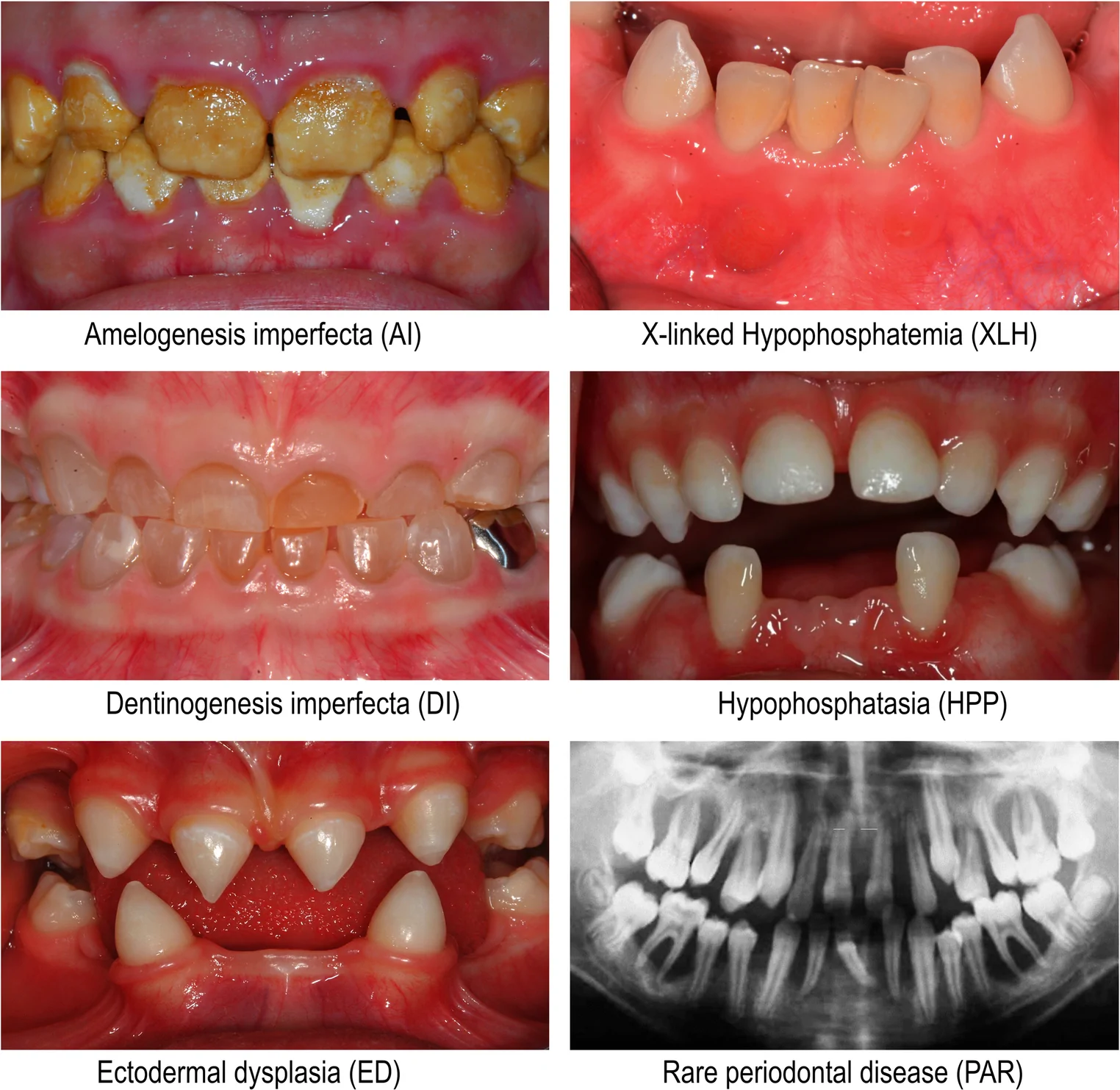
Overview of key clinical and radiological characteristics of the included rare dental disorders

Rare, genetically determined dental disorders may present clinically either as an isolated disorder or as part of a symptom complex in syndromic conditions [1]. They are frequently associated with severe functional impairments, atypical clinical manifestations, and a reduced oral health–related quality of life [2]. The dental management of individuals affected by rare dental disorders presents major challenges for the individuals themselves, their caregivers, general dental practitioners, specialist dentists, and physicians involved in their care and treatment. This is due to both the severity and extent of the clinical findings, which typically manifest in erupting deciduous teeth, affect permanent dentition, and are usually accompanied by functional limitations in old age [3, 4]. The spectrum of clinical presentations (Fig. 1) ranges from the absence of protective tooth enamel (amelogenesis imperfecta, AI) to premature tooth wear (dentinogenesis imperfecta, DI), early tooth loss (e.g., dentinogenesis imperfecta; X-linked hypophosphatemia, XLH; hypophosphatasia, HPP), rapidly progressing periodontitis (e.g., primary immunodeficiencies, PIDs; Papillon–Lefèvre syndrome, PLS), and oligodontia (e.g., ectodermal dysplasia, ED).

**Fig. 2:**
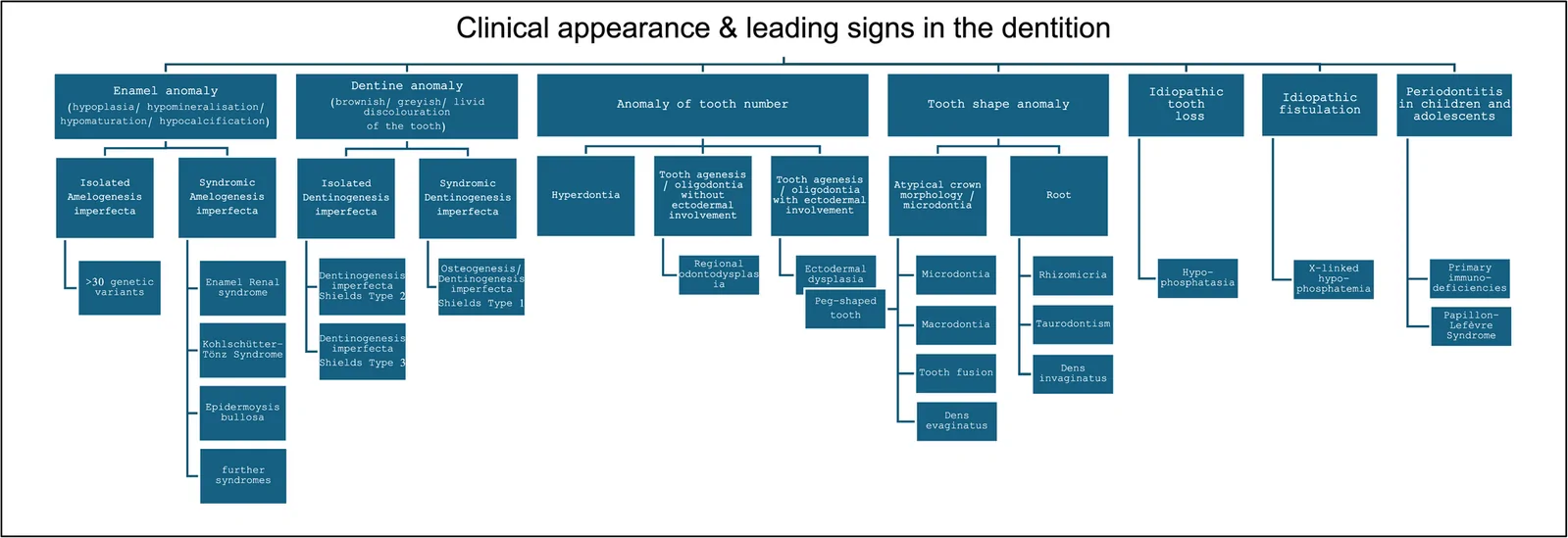
Diagnostic pathway for rare dental disorders

The occurrence of rare dental disorders is caused by genetic abnormalities that are associated with at least functional impairment or loss of function of the encoded protein [1]. This may involve genes directly responsible for the formation of hard dental tissues, such as those involved in amelogenesis imperfecta or dentinogenesis imperfecta. In other cases, a genetic disorder is linked to a primary disease or syndrome in which the teeth form part of the overall clinical picture, as seen in ectodermal dysplasia, X-linked hypophosphatemia, hypophosphatasia, or severe chronic neutropenia [1]. The classification and presentation of rare genetic disorders according to their principal clinical signs provides a straightforward diagnostic framework. Figure 2 provides an overview of the main clinical signs and symptoms, together with the most relevant associated rare dental disorders.

Given the lack of systematically developed recommendations for the complex dental management of individuals with rare dental disorders, the development of a multidisciplinary evidence- and consensus-based guideline was of high clinical relevance. According to the Association of the Scientific Medical Societies in Germany (AWMF; Arbeitsgemeinschaft der Wissenschaftlichen Medizinischen Fachgesellschaften), an S3-level guideline represents the highest level of guideline development, combining systematic evidence appraisal with a structured formal consensus process [5]. The German-language S3-level guideline is publicly available on the AWMF website [6]. The present publication provides an English-language translation and summary of the key evidence- and consensus-based recommendations, aimed at making these accessible to an international readership.

## Methods

The methodology for the development of the German S3-level guideline “Rare dental disorders” [6] is based on the regulations of the AWMF [5]. The methodological details of guideline development, including key questions, PICOS-based search terms, search strategy, risk of bias assessment, and GRADE evaluation, are summarized in the guideline report, which is openly accessible on the AWMF website [7]. Challenges encountered during the literature search have been addressed in a prior publication [8].

In brief, this S3-level guideline was developed between 2021 and 2024. In principle, the guideline development followed the usual methodology and timeline, which requires a mandatory introductory conference to be held following the constitution of the working group. The initial conference took place in Munich in May 2022, to which all contributing authorsand representatives with voting rights were invited. The event was moderated by the AWMF. During this conference, all organisational and thematic aspects were discussed, with a particular focus on the key question: “Which forms of dental care should be part of an age-appropriate, indication-based, and promising long-term treatment for individuals with a rare dental disorder?”. Strategies for literature searches and evidence retrieval were also addressed. The following rare dental disorders were identified as relevant by the working group:

- Amelogenesis imperfecta
- Dentinogenesis imperfecta
- X-linked hypophosphatemia
- Hypophosphatasia
- Ectodermal dysplasia, including oligodontia
- Rare periodontal diseases, e.g., primary immunodeficiencies and Papillon–Lefèvre syndrome

For the systematic literature search, PICOS-based search strategies were created based on the agreed key questions for the different rare diseases. Next, relevant literature databases (MEDLINE via PubMed and Embase) were systematically searched for the period between 1 January 2000 and 31 December 2022. Both systematic reviews and original publications were included in the search; case reports or case series were also taken into account. Additionally, a manual search of German-language dental journals (*Deutsche Zahnärztliche Zeitschrift* and *Oralprophylaxe & Kinderzahnheilkunde*) was conducted to account for relevant literature not indexed in the searched databases. The search results were transferred to the “Rayyan” web application to automatically recognise and remove duplicates [9]. Additionally, title and abstract screening was performed in “Rayyan” based on defined inclusion and exclusion criteria to identify potentially relevant and eligible literature. The full texts of the publications eligible for inclusion were searched, and the relevant information was identified. To assess the quality of evidence, the criteria according to Moga et al. [10] were used in a modified form for case reports and case series. Clinical studies were assessed according to the SIGN criteria [11]. The overall quality of evidence was subsequently rated using the GRADE approach (Grading of Recommendations, Assessment, Development and Evaluation) [12], considering risk of bias, imprecision, indirectness, inconsistency, and publication bias. Given the predominance of case reports and case series in this field, evidence was generally rated as low quality, reflecting the inherent limitations of these study types, such as the absence of control groups and limited proof of efficacy.

The consensus process was methodologically supported by the AWMF and moderated by an independent AWMF-certified medical expert. Based on the reviewed literature, key recommendations were drafted, modified and agreed upon by consensus in the guideline work group. Moreover, the level of recommendation was determined according to the AWMF standards [5]. The final consensus conferences served to discuss, modify and finally vote on the recommendations prepared by the work group in a consensus-oriented manner. Formal consensus was reached at several consensus conferences moderated by the AWMF, to which all contributing authors and representatives with voting rights were invited. The consensus conferences took place as online meetings between December 2023 and February 2024. The executive boards of all the participating academic societies and patient organisations approved the guidelines by June 2024. Upon completion of the project, the guideline was made accessible online in early 2025.

## Evidence- and consensus-based recommendations

The German S3-level guideline contains 8 consensus-based (19.5%) and 33 evidence-based recommendations (80.5%), which are grouped by topic in Tables 1, 2, 3, 4, 5, 6, 7 and 8 [6].

**Table 1:** Evidence- and consensus-based recommendations for the diagnosis of rare dental disorders

Recommendations for all rare dental disorders	Level of evidence (GRADE)	Grade of recommendation
*Recommendation no. 1*: Rare dental disorders can be part of a systemic disease. Diagnosis and lifelong care of affected individuals are complex. These should be carried out with interdisciplinary and, if necessary, interprofessional involvement and should be started at an early stage.	Expert consensus	⇑⇑ Strong
*Recommendation no. 2*: If clinical and radiological findings suggest a genetic cause for a rare dental disorders, genetic diagnostics should be performed, usually in combination with a clinical consultation at a genetic practice or outpatient clinic.	Expert consensus	⇑ Moderate

**Table 2:** Evidence- and consensus-based recommendations for the common management of rare dental disorders

Recommendations for all rare dental disorders	Level of evidence (GRADE)	Grade of recommendation
*Recommendation no. 3*: Individuals affected by rare dental disorders should receive lifelong, risk-oriented and intensive preventive care in order to maintain best possible oral health.	Expert consensus	⇑⇑ Strong
*Recommendation no. 4*: For individuals with extensive treatment needs, dental treatment can be performed under general anaesthesia (e.g. sedation, intubation anaesthesia) if it is not possible by other means.	⊕⊝⊝⊝ Very low	⇔ Open

**Table 3:** Overview of all evidence- and consensus-based recommendations for the dental diagnosis and treatment of dentinogenesis imperfecta (DI)

Recommendations for the management of Dentinogenesis imperfecta	Level of evidence (GRADE)	Grade of recommendation
*Recommendation no. 5*: Direct restorations and prefabricated stainless steel crowns should be used in the primary and mixed dentition to avoid DI-associated loss of dental hard tissues, to compensate for it, and/or to restore the occlusal vertical dimension.	⊕⊝⊝⊝ Very low	⇑⇑ Strong
*Recommendation no. 6*: In cases of reduced occlusal vertical dimension, this should be rehabilitated with prefabricated stainless steel crowns on the primary molars and with either prefabricated stainless steel crowns or individually fabricated indirect single-tooth restorations on first permanent molars.	⊕⊝⊝⊝ Very low	⇑⇑ Strong
*Recommendation no. 7*: Following extraction of a primary tooth, space-maintaining and/or interim prosthetic measures should be undertaken in order to restore masticatory function, support the dentition, and prevent growth-related mesialisation of teeth.	⊕⊝⊝⊝ Very low	⇑ Moderate
*Recommendation no. 8*: Direct restorations and individually fabricated indirect single-tooth crowns should be used in the early permanent dentition to prevent DI-associated loss of dental hard tissue, to compensate for such defects, and/or to stabilise or restore the occlusal vertical dimension. Indirect single-tooth crowns form part of a long-term care strategy.	⊕⊝⊝⊝ Very low	⇑⇑ Strong
*Recommendation no. 9*: The definitive treatment should be implemented starting with the early permanent dentition using the full-mouth rehabilitation approach.	⊕⊝⊝⊝ Very low	⇑⇑ Strong
*Recommendation no. 10*: Individually fabricated indirect single-tooth crowns should be used in posterior teeth of the adult permanent dentition to prevent progressive DI-associated loss of dental hard tissue, to stabilise or restore the occlusal vertical dimension, and to ensure the longevity of anterior restorations.	⊕⊝⊝⊝ Very low	⇑⇑ Strong
*Recommendation no. 11*: Direct restorations and individually fabricated indirect single-tooth crowns should be used in anterior teeth of the adult permanent dentition to compensate for DI-associated loss of dental hard tissue.	⊕⊝⊝⊝ Very low	⇑⇑ Strong
*Recommendation no. 12*: Fixed, implant-supported or removable prosthetic restorations should be used in the adult permanent dentition to compensate for or prevent DI-associated tooth loss, and to stabilise and restore occlusion and masticatory function.	⊕⊝⊝⊝ Very low	⇑⇑ Strong

**Table 4:** Overview of all evidence- and consensus-based recommendations for the dental diagnosis and treatment of amelogenesis imperfecta (AI)

Recommendations for the management of Amelogenesis imperfecta	Level of evidence (GRADE)	Grade of recommendation
*Recommendation no. 13*: Direct restorations, with or without the use of matrices, should be placed in anterior and posterior teeth of the primary and mixed dentitions to achieve pain relief, restore function, and improve aesthetics.	⊕⊕⊝⊝ Low	⇑⇑ Strong
*Recommendation no. 14*: Prefabricated stainless steel crowns should be placed on primary molars at an early stage to restore tooth form and occlusion.	⊕⊕⊝⊝ Low	⇑⇑ Strong
*Recommendation no. 15*: Prefabricated stainless steel crowns or individually fabricated indirect single-tooth restorations should be placed on permanent molars at an early stage to restore tooth form and occlusion.	⊕⊕⊝⊝ Low	⇑ Moderate
*Recommendation no. 16*: Individually fabricated indirect single-tooth restorations should be placed on permanent anterior teeth already in the mixed dentition to achieve pain relief, aesthetics, and physiological function.	⊕⊕⊝⊝ Low	⇑ Moderate
*Recommendation no. 17*: Orthodontic or combined orthodontic-surgical treatments of malocclusion should be initiated according to its indication.	⊕⊝⊝⊝ Very low	⇑ Moderate
*Recommendation no. 18*: Individually fabricated indirect single-tooth crowns should preferably be used, beside direct restorations, for the long-term achievement of pain relief, aesthetics, and physiological function, as indicated.	⊕⊕⊝⊝ Low	⇑⇑ Strong
*Recommendation no. 19*: Individually fabricated indirect single-tooth crowns should be placed in the mixed or early permanent dentition, following tooth eruption and appropriate adjustment of the occlusal relationship, to achieve pain relief, aesthetics, and physiological function. This is part of a long-term, interdisciplinary management strategy.	⊕⊕⊝⊝ Low	⇑⇑ Strong
*Recommendation no. 20*: Definitive treatment should begin in the early permanent dentition using the concept of full-mouth rehabilitation.	⊕⊕⊝⊝ Low	⇑⇑ Strong
*Recommendation no. 21*: Definitive, individually fabricated indirect single-tooth crowns should be placed in the permanent dentition to achieve pain relief, aesthetics, and physiological function.	⊕⊕⊕⊝ Moderate	⇑⇑ Strong

**Table 5:** Overview of all evidence- and consensus-based recommendations for the dental diagnosis and treatment of X-linked hypophosphatemia (XLH)

Recommendations for the management of X-linked hypophosphatemia	Level of evidence (GRADE)	Grade of recommendation
*Recommendation no. 22*: During dental examinations at six-month intervals from the eruption of the first tooth, attention should be given to symptoms of pulp inflammation or necrosis-such as pain, discolouration, fistulae, swelling, or abscesses-in caries-free teeth without prior trauma. Diagnostic procedures should, if necessary, be supplemented by sensitivity testing and radiographic examination.	⊕⊕⊕⊝ Moderate	⇑⇑ Strong
*Recommendation no. 23*: To prevent pulpal infection and further loss of hard tissue, exposed coronal dentine should be sealed adhesively and carious lesions should be treated at an early stage.	Expert consensus	⇑⇑ Strong
*Recommendation no. 24*: To minimise the risk of exposing extended pulp horns or large pulp chambers, hard tissue–reducing procedures (tooth preparations) should be performed in a minimally invasive manner.	Expert consensus	⇑⇑ Strong
*Recommendation no. 25*: In the primary dentition, teeth affected by XLH that are nonvital, having fistulae associated with apical periodontitis, mobile, or causing pain should be extracted.	Expert consensus	⇑⇑ Strong
*Recommendation no. 26*: In the permanent dentition, nonvital teeth in individuals with XLH should undergo endodontic treatment in order to preserve the teeth.	⊕⊝⊝⊝ Very low	⇑ Moderate

**Table 6:** Overview of all evidence- and consensus-based recommendations for the dental diagnosis and treatment of hypophosphatasia (HPP)

Recommendations for the management of Hypophosphatasia	Level of evidence (GRADE)	Grade of recommendation
*Recommendation no. 27*: In cases of early, ideopathic tooth loss, particularly in the primary dentition, medical diagnostic evaluation for hypophosphatasia should be initiated.	Expert consensus	⇑⇑ Strong
*Recommendation no. 28*: In the primary dentition, loose teeth causing pain in individuals with hypophosphatasia should be extracted.	Expert consensus	⇑⇑ Strong

**Table 7:** Overview of all evidence- and consensus-based recommendations for the dental diagnosis and treatment of ectodermal dysplasia (ED), including oligodontia

Recommendations for for the management of Ectodermal dysplasia and oligodontia	Level of evidence (GRADE)	Grade of recommendation
*Recommendation no. 29*: Paediatric dentures should be fabricated for children with oligo-/anodontia in the primary and mixed dentitions to establish or maintain function and aesthetics, once sufficient cooperation is achievable.	⊕⊝⊝⊝ Very low	⇑ Moderate
*Recommendation no. 30*: Paediatric dentures should be regularly reviewed during dental check-ups with regard to growth-related fit, function, and aesthetics, and adjusted or remade as necessary.	⊕⊝⊝⊝ Very low	⇑⇑ Strong
*Recommendation no. 31*: Direct composite restorations should be used to reshape microdont anterior teeth.	⊕⊝⊝⊝ Very low	⇑ Moderate
*Recommendation no. 32/A*: In individuals with oligodontia, treatment of the early permanent dentition should be planned in such a way that, after completion of growth, the broadest possible spectrum of definitive treatment options can be offered and implemented. *Recommendation no. 32/B*: In the early permanent dentition, functionally high-quality care with a high level of social acceptability should already be provided.	⊕⊝⊝⊝ Very low	⇑ Moderate
*Recommendation no. 33*: An individual, comprehensive prosthetic and/or implant-supported prosthetic treatment should be provided for individuals with oligodontia as definitive therapy to restore function and aesthetics.	⊕⊝⊝⊝ Very low	⇑⇑ Strong
*Recommendation no. 34*: Orthodontic and/or surgical treatment should be carried out as indicated to optimise jaw relations, tooth position and distribution in preparation for prosthetic and/or implant-supported prosthetic rehabilitation.	⊕⊝⊝⊝ Very low	⇑ Moderate
*Recommendation no. 35*: Implant treatment should be provided in accordance with the S3 guideline “Dental implant therapy in cases of multiple tooth agenesis and syndromes“ [16]	⊕⊝⊝⊝ Very low	⇑⇑ Strong

**Table 8:** Overview of all evidence- and consensus-based recommendations for the dental diagnosis and treatment of periodontitis as a manifestation of a rare systemic disease

Recommendations for periodontitis as a manifestation of a rare systemic disease	Level of evidence (GRADE)	Grade of recommendation
*Recommendation no. 36*: Treatment of periodontitis as a manifestation of a rare systemic disease should, where indicated, be accompanied by systemic therapy of the underlying disease.	⊕⊝⊝⊝ Very low	⇑⇑ Strong
*Recommendation no. 37*: Treatment of periodontitis as a manifestation of a rare systemic disease should follow the S3-level guidelines “Treatment of periodontitis stages I–III“ (083 − 043) and “Treatment of periodontitis stage IV“ (083 − 056). [13,14]	⊕⊝⊝⊝ Very low	⇑⇑ Strong
*Recommendation no. 38*: Treatment of periodontitis as a manifestation of a rare systemic disease may include adjunctive administration of antibiotics in conjunction with subgingival instrumentation (first choice: amoxicillin and metronidazole).	⊕⊝⊝⊝ Very low	⇔ Open
*Recommendation no. 39*: In periodontitis as a manifestation of a rare systemic disease, strict home-based and professional biofilm management should be implemented, if necessary, with the use of antiseptics such as chlorhexidine.	⊕⊝⊝⊝ Very low	⇑⇑ Strong
*Recommendation no. 40*: Treatment of periodontitis as a manifestation of a rare systemic disease should not differ between the primary and permanent dentitions.	⊕⊝⊝⊝ Very low	⇑ Moderate
*Recommendation no. 41*: Individuals with severe periodontitis as a manifestation of a rare systemic disease should be informed about the increased risk of implant failure.	⊕⊝⊝⊝ Very low	⇑⇑ Strong

### Dental diagnostics of rare dental disorders

The clinical diagnosis and classification of rare dental disorders are usually based on visual assessment by an experienced dentist (Fig. 1). The key diagnostic indicators include the clinical appearance of the teeth, their morphology, the number of teeth, and thus atypical findings that deviate significantly from the norm (Fig. 2). In rare dental disorders, all teeth in the primary and permanent dentition may appear nearly identical. This is particularly the case for DI, AI, or most rare periodontal diseases. In systemic diseases for which drug therapy is available, such as HPP, XLH, or severe chronic neutropenia, the extent of dental involvement varies depending on the onset and duration of therapy. In oligodontia or ED, different degrees of hypodontia are observed. Hence, rare dental disorders are associated with more or less generalised involvement of the entire dentition. The clinical signs can usually be detected during routine clinical examination, whereas radiographs taken according to indications provide additional diagnostic information. With respect to molecular background, it should be emphasised that only genetic analyses can provide precise information about the aetiology and prognosis of the disease, potential complications, and possible involvement of other organ systems.

### Genetic diagnostics

Numerous rare disorders have a specific monogenic or chromosomal origin. Important indicators of a genetically determined dental disorder include an unusual and/or generalised constellation of symptoms, primary teeth with atypical shape or structure, multisystem disease or a syndromic clinical pattern, and the occurrence of unusual and/or similar findings in several family members. If a genetic disease is suspected, the family history should be carefully assessed. This entails identifying similar symptoms in siblings, parents, grandparents, and other relatives across several generations. In some families, the observed phenotypes in the pedigree provide evidence for a specific inheritance pattern (e.g., autosomal dominant, autosomal recessive, or X-linked). The disease itself, as well as carriers of the trait, are sometimes already known in a family, and the individual concerned may present to the dental practice with a suspected diagnosis. Referral to a medical genetic outpatient clinic or specialist practice is prudent for the clinical clarification of a suspected diagnosis and as a prerequisite for targeted genetic diagnostics. If a primary genetic aetiology is suspected, the dental examination should be complemented by genetic analysis, with the aim of establishing a precise molecular diagnosis. This requires genetic counselling and the consent of the tested individual [4]. A suspected monogenic disorder is usually investigated via massively parallel sequencing, whereas a chromosomal (structural genomic) disorder is primarily assessed via (molecular) cytogenetic testing. In many cases, genetic diagnosis provides essential information regarding the clinical manifestation and prognosis of the disease, potential complications, and possible involvement of other organ systems. Such information is often a decisive factor in the selection of appropriate treatment options. Sometimes variants of uncertain significance (VUS) are identified that may or may not be the explanation for the clinical manifestation. In this case, the result must not be regarded as diagnostic, and further investigations in the family or functional research tests may be required to clarify the situation. It is also important to note that an unremarkable genetic finding does not exclude a genetic aetiology. If a genetically determined dental disease is confirmed, genetic counselling is indicated.

### General clinical recommendations

The need for sedation or treatment under general anaesthesia for conservative–surgical restoration of dentition has been documented, particularly in children and adolescents affected by rare dental disorders. The aim of this approach is to enable dental care in cases where the child’s ability to cooperate is not (yet) sufficient. The principal indications for tooth restoration under general anaesthesia are extensive treatment requirements, such as multiple restorations and/or extractions, which cannot be managed through stepwise treatment with or without local anaesthesia (Table 2).

In addition, the therapeutic approach of (re)establishing function and aesthetics across the entire dentition should often be considered when generalised findings compromise normal occlusion, function, and aesthetics. As rare dental disorders are typically associated with changes affecting all primary and permanent teeth equally, the concept of full-mouth rehabilitation constitutes an integral part of the dental management strategy in many cases. This includes the preservation of restorable primary and permanent teeth, as well as the restoration of tooth morphology, occlusion, and aesthetics. The concept is applied across all age groups with the use of various restorative techniques (Table 2).

A rare dental disorder is usually determined by a specific genotype, which is associated with a characteristic pathophysiological and clinical manifestation (phenotype) and, ultimately, individual treatment options. As the phenotype may vary considerably, dental care should always be tailored to the patient’s individual needs. Furthermore, dental treatment differs according to the stage of dentition development (primary, mixed, early permanent, or adult dentition). This necessitates age specific, disease-adapted treatment strategies.

### Dentinogenesis imperfecta (DI)

Taking into account the respective indications, both direct and indirect forms of restorations are generally capable of compensating for DI-associated tooth structure defects and restoring the shape, function, and aesthetics of the affected teeth with a high degree of reliability. Case reports indicate that definitive restoration of the entire permanent dentition is usually undertaken towards the end of the second or the beginning of the third decade of life. From a clinical–practical perspective, indirect restorations are not ad hoc procedures but require strategic planning, considering the growth and development of the dentition. The primary objective is to secure or restore functionality and the vertical occlusal dimension in the posterior region, after which the indicated restorations of the anterior teeth can be carried out. This process of full-mouth rehabilitation begins in childhood and adolescence and is completed only once growth has ceased in adulthood, while lifelong restorative needs may remain. Such treatment necessitates a conceptualised approach to dental care for individuals with DI, with the aim of preserving their natural dentition (Table 3).

### Amelogenesis imperfecta (AI)

Given the hypomineralised and/or hypoplastic enamel, indirect restoration techniques represent the treatment of choice in the permanent dentition for addressing AI-associated pain and hypersensitivity, as well as the aesthetic and functional limitations resulting from enamel defects. Such treatment is associated with great health benefits and leads to substantial improvements in both oral health-related and social quality of life for individuals with AI. From a clinical–practical perspective, indirect restorations require early and systematic planning, beginning with the eruption of the first permanent teeth. Particular attention should be given to achieving stable function in the posterior region, followed by definitive restoration of anterior teeth, to harmonise function and aesthetics over the long term (Table 4).

### X-linked hypophosphatemia (XLH)

In individuals with XLH, root canal treatment of permanent teeth is an appropriate option for managing pulp necrosis and thereby preserving the affected tooth. This approach enables the preservation of essential orofacial functions and helps to avoid cost-intensive tooth replacement procedures. Where tooth preservation is not feasible by endodontic means, prosthetic interventions are indicated to replace missing teeth. Implants should be considered (Table 5).

### Hypophosphatasia (HPP)

The main clinical symptom of most HPP subtypes is the premature loss of primary teeth in early childhood. In patients without other clinical abnormalities, this symptom can enable the dentist to make a decisive contribution to initiating targeted diagnostics. Specific laboratory diagnostics can confirm suspected HPP. Loosened teeth are usually not preserved. In primary dentition, consideration should be given to whether space maintainers are appropriate (Table 6). This should always be undertaken within the framework of regular follow-up examinations to monitor disease progression, oral function, and the stability of prosthetic treatment.

### Ectodermal dysplasia (ED), including oligodontia

The main clinical features of ED are multiple missing primary and permanent teeth, along with characteristic dermal manifestations. These typically include dry and fragile skin, fine and sparse hair, nail dystrophies, and reduced or absent function of the sweat, sebaceous, and salivary glands. ED thus represents a symptom complex that requires interdisciplinary management. To compensate for dental deficits, prosthetic replacement of missing teeth is usually indicated, taking into account the jaw and occlusal development. In addition, accurate impressions of microdontic teeth must be reliably obtained. The treatment options considered are established and evidence-based dental measures (Table 7).

### Rare periodontal diseases, e.g., primary Immunodeficiencies (PID) and Papillon-Lefèvre-Syndrome (PLS)

n addition to the principal clinical manifestation of rapidly progressive periodontitis with onset in childhood or adolescence, other organs or functional systems may also be affected. This necessitates referral for further medical diagnostics and management (e.g., human genetics, paediatrics, or internal medicine), if this has not already been undertaken prior to the dental findings. Systemic therapy appears to exert a decisive influence on the outcome of periodontal treatment, particularly in the presence of immunodeficiencies. In patients with severe chronic neutropenia, treatment with granulocyte colony-stimulating factor (G-CSF) is indicated because it is a life-threatening condition. In addition to a reduced number of neutrophil granulocytes, their function may also be impaired. If systemic therapy exists that restores this function completely or partially, it should be initiated. Periodontitis as a disease manifestation can be prevented or at least significantly delayed. In addition, rigorous periodontal therapy remains the only established approach for potential tooth preservation. No alternative to conventional periodontal therapy is currently known (Table 8).

## Discussion

With regard to the main objective of this guideline project, namely, to describe rare genetic dental diseases, it can be stated that common dental conditions have been identified and mapped. This has made it possible to consolidate diverse entities and to avoid complex guideline processes for each individual rare dental disorder. Moreover, this approach allows additional conditions to be incorporated in the course of regular updates, as addressing hundreds of rare dental disorders [1] in separate guideline projects would be impossible.

The prevention of secondary conditions such as caries, gingivitis, or periodontitis is a high priority in the dental management of rare dental disorders. This is particularly important, as primary prevention is generally not possible, and the objective is to avoid further deterioration of the underlying disorder. Preventive measures should include dietary counselling to reduce sugar intake, optimal oral hygiene, professional tooth cleaning, and appropriate fluoride application (Table 2). Restorative management of teeth is particularly essential in cases of AI and DI (Tables 2 and 3) to prevent loss of tooth substance and loss of the vertical occlusal dimension in the primary and mixed dentition. Evidence-based approaches such as direct restorations play a key role in both dentition and across all age groups. In primary dentition, prefabricated stainless-steel crowns provide a reliable single-visit option for the restoration of posterior teeth. Direct restorations are also suitable for the recontouring of microdontic anterior teeth in individuals with ED (Table 4). Furthermore, space-maintaining or interim prosthetic measures should be taken following extraction of the primary dentition to maintain masticatory function and prevent tooth migration and tipping. The fabrication of paediatric prostheses, along with their regular monitoring and adjustment, constitutes an essential element of dental management in cases of tooth agenesis in the primary and mixed dentition (Table 4). Premature tooth loss may also occur in cases of AI or DI and likewise requires prosthetic compensation. Non-vital teeth with spontaneously occurring dental infections in individuals with XLH, as well as loose teeth in those with HPP, can also result in early tooth loss (Tables 5 and 6). Across all age groups, from the eruption of the first tooth onwards, particular attention should be given to individuals with XLH to signs of pulpal inflammation or necrosis in caries-free teeth without previous trauma. The exposed coronal dentine should be sealed adhesively, and carious lesions should be treated at an early stage. Due to enlarged pulp horns, any removal of tooth substance should be carried out via minimally invasive techniques wherever possible. The available data suggest that patients suffering from XLH or HPP benefit from well-adjusted systemic therapy when dental treatments affecting the alveolar bones are performed, such as orthodontic, periodontal, and implantological treatments. More time should be allowed for bone remodelling in orthodontics and implantology by extending the duration of treatment. (Table 5).

Prosthetic therapy in the sense of full-mouth rehabilitation plays a pivotal role in the management of AI and DI. Beginning in adolescence, indirectly fabricated restorations should be placed on the first permanent molars to prevent further loss of tooth substance, ensure pain control, and maintain the vertical occlusal dimension. This therapeutic approach is the basis for the long-term rehabilitation of the entire permanent dentition (Tables 2 and 3). In ED individuals, functional high-quality prosthetic rehabilitation should likewise be initiated in the early permanent dentition. Prosthetic treatment planning should aim to provide different treatment options once growth has been completed and to successfully implement the individually indicated definitive intervention (Table 4). For the replacement of permanent teeth, fixed, implant-supported, or removable prosthetic solutions are available, with the objective of compensating for tooth loss and restoring masticatory function [13].

The treatment of periodontitis as a manifestation of a rare systemic disease should, where appropriate, be complemented by systemic management of the underlying condition. The therapeutic approach does not differ between primary and permanent dentitions and follows the current clinical practice guidelines for periodontitis stages I–III and IV [14, 15] (Table 8). Adjunctive antibiotic therapy can be administered at the same time as subgingival instrumentation. Strict home-based and professional biofilm management, if necessary supported by antiseptics, is essential. Individuals with severe periodontitis should additionally be informed of the increased risk of implant failure (Table 8).

Worldwide, individuals affected by rare genetic dental diseases continue to face significant barriers to dental care. This is remarkable given that the UN Convention on the Rights of Persons with Disabilities stipulates that persons with special needs are entitled to the highest attainable standard of health and guaranteed access to health rehabilitation [16]. In practice, however, the costs associated with treatment are often considerable, and depending on the healthcare system, not all measures can be ensured or reimbursed. The financial burden is particularly pronounced in cases of generalised findings, where direct restorations, endodontic treatment, prosthetic rehabilitation, or implant-supported solutions may be required repeatedly throughout life. For affected individuals and their families, these expenses represent a substantial and often unsustainable burden. The complexity of these conditions therefore calls for low-level and lifelong access to appropriate diagnostic, preventive, and rehabilitative services to mitigate disadvantages and ensure the highest possible degree of participation in all aspects of life.

From a methodological perspective, the scientific literature and database on rare dental disorders are insufficient. Clinical studies were documented almost exclusively for AI, whereas for all other conditions, only case reports or case series were available. In addition, there were no standardised endpoints for the respective treatment options, which made it difficult to classify the case reports and series. This likely reflects the typical situation in which very few individuals with rare dental disorders are diagnosed and treated within different dental care settings, making it unsurprising that the required sample sizes for clinical trials can hardly, if at all, be recruited. The available case reports and series also varied in terms of observation periods and were highly heterogeneous in quality [8]. Overall, the literature indicates that the evidence base for rare dental disorders is limited and generally of low quality. Moreover, the number of available publications differed considerably among the selected diseases: most publications, predominantly case reports and series, were found for AI, whereas XLH and HPP were the least frequently reported. These differences in frequency may indirectly reflect the prevalence of these conditions in the population.

## Conclusion

The recently published German S3-level guideline “Rare Dental Disorders” contains a total of 41 recommendations for diagnosis and therapy. It thus provides dentists, affected individuals, and all other stakeholders with up-to-date recommendations for the highest possible quality of individualised dental care. The applicability of these recommendations across different healthcare systems may vary depending on the availability of specialist expertise, genetic diagnostic services, and financial resources. Future research should prioritise standardised reporting of clinical outcomes and genotype-phenotype correlation analyses to strengthen the evidence base for the dental management of rare genetic dental disorders. The guideline will be subject to regular review and update in accordance with AWMF standards.

## Data Availability

No datasets were generated or analysed during the current study.

## Declarations

### Participating organisations and their representatives

The guideline was coordinated by the German Society for Paediatric Dentistry (DGKiZ, Ina Schüler), the German Society for Prosthetic Dentistry and Biomaterials (DGPro, Daniel Edelhoff), the German Society for Computer-Aided Dentistry (DGCZ, Jan-Frederik Gueth), the German Society for Human Genetics (GfH, Johannes Zschocke), and the German Society for Dental, Oral and Maxillofacial Medicine (DGZMK, Julia Winter).

The authors gratefully acknowledge the following professional societies, organisations, and their representatives for their contributions:, which participated in the guideline development process: Working Group on Paediatric Immunology (API, Fabian Hauck); German Society of Implantology (DGI, Henrik Terheyden); German Society of Orthodontics (DGKFO, Callin Jacobs); German Society of Paediatrics and Adolescent Medicine (DGKJ, Helge Hebestreit); German Society of Oral and Maxillofacial Surgery (DGMKG, Sven Otto); German Society of Osteology (DGO, Ralf Oheim); Society of Paediatric Radiology (GPR, Hans-Joachim Mentzel); German Society of Periodontology (DG PARO, Peter Eikholz); German Society of Dental Implantology (DGZI, Arzu Tuna); German Society of Conservative Dentistry (DGZ, Reinhard Schilke); Society of Neuropaediatrics (GNP, Heike Kölbel, Regina Trollmann); German Society of Dentistry for Individuals with Disabilities or Special Medical Needs (DGZMB, Peter Schmidt); Working Group on Oral and Maxillofacial Surgery (AGOKi, Andreas Schön); Professional Association of German Oral Surgeons (BDO, Frank Strietzel); Federal Association of Implantologists in Germany (BDIZ EDI, Jörg Neugebauer); Federal Association of Paediatric Dentists (BuKiZ, Monika Prinz-Kattinger); Professional Association of Paediatricians (BVKJ, Burkhard Lawrenz); Federal Association of Dentists in Public Health Services (BZÖG, Silke Riemer); German Society of Aesthetic Dentistry (DGÄZ, Robert Sader); German Society of Endodontology and Dental Traumatology (DGET, Anahita Jablonski-Momeni); German Society of Laser Dentistry (DGL, Gabriele Schindler-Hultzsch); German Society of Preventive Dentistry (DGPZM, Jochen Jackowski); German Society of Restorative and Regenerative Dentistry (DGR^2^Z, Matthias Widbiller); European Reference Network on Rare Craniofacial Anomalies and ENT Disorders (ERN Cranio, Sigmar Reinert); Free Association of German Dentists (FVDZ, Thomas Wolf); Association of Medical Professions (VMF, Marion Schellmann, Sylvia Gabel).

The following patient advocacy organisations were represented in the guideline development process: German Osteogenesis Imperfecta Society (Deutsche Gesellschaft für Osteogenesis imperfecta Betroffene e.V., Claudia Finis); Ectodermal Dysplasia Society Germany (Ektodermale Dysplasie e.V., Andrea Burk); Parent Initiative for Apert Syndrome and Related Malformations (Elterninitiative Apert-Syndrom und verwandte Fehlbildungen e.V., Sandra Mösche); Hypophosphatasia Germany (Hypophosphatasie Deutschland e.V., Gerald Brandt); X-linked Hypophosphatemia Germany (Phosphatdiabetes e.V., Jana Bombosch, Martha Kirchhoff).

We would like to express our gratitude to Dr. Monika Nothacker (AMWF) for providing invaluable methodological advice during the development of this guideline project. Her expertise and guidance were instrumental in the development of the guideline. The working group thanks Dr. Anke Weber (DGZMK) and Dr. Birgit Marré (DGZMK) for their organisational and methodological support.
